# Serotype M3 and M28 Group A Streptococci Have Distinct Capacities to Evade Neutrophil and TNF-α Responses and to Invade Soft Tissues

**DOI:** 10.1371/journal.pone.0129417

**Published:** 2015-06-05

**Authors:** Zachary W. Stetzner, Dengfeng Li, Wenchao Feng, Mengyao Liu, Guanghui Liu, James Wiley, Benfang Lei

**Affiliations:** Department of Microbiology and Immunology, Montana State University, Bozeman, Montana 59718, United States of America; Centers for Disease Control & Prevention, UNITED STATES

## Abstract

The M3 Serotype of Group A Streptococcus (GAS) is one of the three most frequent serotypes associated with severe invasive GAS infections, such as necrotizing fasciitis, in the United States and other industrialized countries. The basis for this association and hypervirulence of invasive serotype M3 GAS is not fully understood. In this study, the sequenced serotype M3 strain, MGAS315, and serotype M28 strain, MGAS6180, were characterized in parallel to determine whether contemporary M3 GAS has a higher capacity to invade soft tissues than M28 GAS. In subcutaneous infection, MGAS315 invaded almost the whole skin, inhibited neutrophil recruitment and TNF-α production, and was lethal in subcutaneous infection of mice, whereas MGAS6180 did not invade skin, induced robust neutrophil infiltration and TNF-α production, and failed to kill mice. In contrast to MGAS6180, MGAS315 had *covS* G1370T mutation. Either replacement of the *covS* ^1370T^ gene with wild-type *covS* in MGAS315 chromosome or *in trans* expression of wild-type *covS* in MGAS315 reduced expression of CovRS-controlled virulence genes *hasA*, *spyCEP*, and *sse* by >10 fold. MGAS315 *covS* ^wt^ lost the capacity to extensively invade skin and to inhibit neutrophil recruitment and had attenuated virulence, indicating that the *covS* G1370T mutation critically contribute to the hypervirulence of MGAS315. Under the background of functional CovRS, MGAS315 *covS* ^wt^ still caused greater lesions than MGAS6180, and, consistently under the background of *covS* deletion, MGAS6180 Δ*covS* caused smaller lesions than MGAS315 Δ*covS*. Thus, contemporary invasive M3 GAS has a higher capacity to evade neutrophil and TNF-α responses and to invade soft tissue than M28 GAS and that this skin-invading capacity of M3 GAS is maximized by natural CovRS mutations. These findings enhance our understanding of the basis for the frequent association of M3 GAS with necrotizing fasciitis.

## Introduction

Group A *Streptococcus* (GAS) is a major human pathogen that commonly causes pharyngitis and superficial skin infections [1]. Historically, GAS caused severe and frequent epidemics of invasive and often fatal illnesses in the 19th century [2]. While the number of severe invasive GAS infections declined in the majority of the 20^th^ century, cases of streptococcal necrotizing fasciitis, toxic shock syndrome, and bacteremia have significantly increased in industrialized countries since the 1980s. Contemporary severe invasive GAS infections are caused disproportionately by serotype M1, M3, and M12 GAS [3–6]. Patients with severe invasive infections caused by serotype M3 strains are more likely to have necrotizing fasciitis than patients with invasive infections caused by other serotype strains, and invasive M3 GAS causes a higher mortality rate than invasive strains of other serotypes [7]. Invasive GAS isolates often display higher capacity to invade skin tissue, to evade neutrophil responses, and to kill animals in animal models of necrotizing fasciitis than pharyngitis GAS isolates [8,9]. Extensive investigation has been conducted to understand the emergence of invasive GAS clones and the basis for their hypervirulence. Two sequenced invasive M3 strains, MGAS315 and SSI-1, are genetically representative of the principal clone of M3 isolates that cause contemporary invasive infections in the U.S., Canada, Western Europe, and Japan [10,11]. A prophage, Φ315.4, in the sequenced strains encode the superantigen, SpeK, and phospholipase A2, SlaA, and these two genes were not present in serotype M3 GAS strains recovered before 1987 but were present in invasive M3 isolates recovered after 1987 [10]. Pharyngitis and invasive M3 GAS groups are highly related to each other and belong to the same genetic pool [12]. However, pharyngitis and invasive groups of M3 GAS show distinct patterns of diversifying selection and have more polymorphisms in the hyaluronic acid capsule synthesis operon and in the *covS* gene of the two-component regulatory system CovRS (also known as CsrRS), respectively [12]. Whether the frequent *covS* polymorphisms among invasive M3 GAS isolates critically contribute to their hypervirulence has not been experimentally demonstrated, and the basis for the hypervirulence of invasive M3 GAS is not fully understood.

CovRS negatively regulates multiple virulence factors [13–16], including those involved in innate immune evasion, such as the capsule synthase HasA [13], IL-8/CXC chemokine peptidase SpyCEP [17], platelet-activating factor acetylhydrolase SsE [18], and DNase Sda1 [19]. Natural CovRS mutants of a M1T1 clone of serotype M1 GAS have a high capacity to cause skin invasion, innate immune evasion, and systemic dissemination and thus have hypervirulence [19–22]. Natural CovRS mutations of the M1T1 clone of M1 GAS are a critical factor for the association of serotype M1 GAS with severe invasive GAS infections since the 1980s. The M1T1 clone emerged in the 1980s and has globally disseminated since then [23–28]. The M1T1 clone is believed to be evolved from a M1 progenitor by the acquisitions of DNase Sda1 (also known as SdaD2)- and superantigen SpeA-encoding prophages and by horizontal gene transfer of a 36-kb chromosomal region encoding toxins NAD^+^-glycohydrolase and streptolysis O [29,30]. A number of studies by several groups support a model for the occurrence of hypervirulent M1T1 GAS in which neutrophils select spontaneous CovRS mutations of the M1T1 GAS that enhance the expression of a subset of virulence genes and maximize the potential of GAS to evade neutrophil responses, resulting in M1T1 GAS variants with enhanced survival and hypervirulence [19–22,31–37].

The basis for the frequent association of serotype M3 GAS with severe invasive infections, especially necrotizing fasciitis, is not well understood. One question is whether contemporary M3 GAS is more potent in invading soft tissues than GAS of many other serotypes. Another question is whether CovS mutations, like in M1T1 GAS, critically contribute to hypervirulence of invasive M3 GAS. The sequenced M3 GAS strain, MGAS315, which was isolated in Texas, is highly virulent in a mouse model of intraperitoneal infection [38] and has a G-to-T mutation at base 1370 of the *covS* gene (*covS* ^G1370T^) that results in the Gly-to-Val alteration at residue 457 of the CovS protein (CovS^G457V^) [39]. This *covS* G1370T mutation is present in 31 of 95 invasive serotype M3 isolates that were recovered between January 1992 and December 2007 in Ontario, Canada [39]. It should be noted that CovS^457V^ was considered as the wild-type CovS in the analysis by Beres et al. [39]. While it is reasonable to assume that natural CovRS mutations enhance virulence of GAS, a lot of CovRS mutations, especially CovS mutations in M3 GAS including the CovS^G457V^ mutation, have not experimentally shown to critically contribute to hypervirulence. This study is designed to address these questions. We compared the phenotype of M3 strain MGAS315, the sequence serotype M28 strain MGAS6180, and their derivatives in subcutaneous mouse infection and virulence gene expression. Results indicate that GAS strains with the MGAS315 background had greater capacity to invade soft tissue than GAS strains with the MGAS6180 background in both the presence and absence of functional CovRS. Furthermore, the CovS G457V or *covS* G1370T mutation greatly enhances the capacity of MGAS315 to invade soft tissues.

## Materials and Methods

### Declaration of ethical approval

All animal experimental procedures were carried out in strict accordance with the recommendations in the Guide for the Care and Use of Laboratory Animals of the National Institutes of Health [40]. The protocols for the experiments were approved by the Institutional Animal Care and Use Committee at MSU (Permit numbers: 2011–57 and 2014–45).

### Bacterial strains and growth

Serotype M3 GAS strain MGAS315 [10] and serotype M1 strain MGAS2221 [16] have been described, and serotype M28 strain MGAS6180 [41] was obtained from American Type Culture Collection (ATCC No. BAA1064). These strains and their derivative strains (Table 1) were grown in Todd-Hewitt broth supplemented with 0.2% yeast extract (THY) and with 10 mg chloramphenicol per liter when it was needed. GAS bacteria at the exponential growth phase were harvested by centrifugation and washed three times with pyrogen-free Dulbecco's phosphate-buffered saline (DPBS) and resuspended in DPBS. Inocula were determined by plating.

**Table 1:** Group A Streptococcus strains and plasmids used in this study.

Strain or plamid	Description	Reference
MGAS6180	Sequenced serotype M28 strain	[41]
MGAS6180 Δ*covS*	A *covS* deletion mutant of MGAS6180	[42]
MGAS6180/pDCBB-*covS* ^wt^	MGAS6180 carrying pDCBB-*covS* ^wt^	This study
MGAS6180/pDCBB-*ropB*	MGAS6180 carrying pDCBB-*ropB*	This study
MGAS315	Serotype M3 strain with *covS* ^1370T^	[10]
MGAS315 Δ*covS*	A *covS* deletion mutant of MGAS315	This study
MGAS315 *covS* ^wt^	A strain from knocking in wt *covS* into MGAS315 Δ*covS*	This study
MGAS315/pDCBB-*covS* ^wt^	MGAS315 carrying plasmid pDCBB-*covS* ^wt^	This study
MGAS315 *covS* ^wt^:: p740-Δ*covS*	Strain from insertion of p740-Δ*covS* into the *covRS* locus of MGAS315 *covS* ^wt^	This study
MGAS2221	M1T1 GAS strain	[16]
MGAS2221 Δ*covS*	A *covS* deletion mutant of MGAS2221	[16]
MGAS2221 Δ*covS*/pDCBB-*covS* ^wt^	MGAS2221 Δ*covS* carrying pDCBB-*covS* ^wt^	This study
MGAS2221 *ΔcovS*/pDCBB-*covS* ^1370T^	MGAS2221 Δ*covS* carrying pDCBB-*covS* ^1370T^	This study
pDCBB-*covS* ^wt^	MGAS2221 *covS* cloned into pDCBB	This study
pDCBB-*covS* ^1370T^	MGAS315 *covS* ^1370T^ cloned into pDCBB	This study
p740-Δ*covS*	Suicide plasmid to delete *covS*	[22]
p740-*covS* ^wt^	Suicide plasmid to knock in wt *covS*	[22]
pDCBB-*ropB*	wt *ropB* cloned into pDCBB	This study

### Generation of MGAS315 Δ*covS*, MGAS315 *covS* ^wt^, and MGAS315 *covS* ^wt^::p740-Δ*covS*

A 1,290-bp internal fragment of the *covS* ^G1370T^ gene in MGAS315 was deleted using the suicide plasmid p740-Δ*covS* (Table 1), which was previously referred as pΔ*covS* ^Δ1bp^ [22]. The deletion procedure to obtain a MGAS315 Δ*covS* strain was essentially same as that in the generation of a Δ*covS* mutant of MGAS5005 [22]. The same procedure was used to knock in the wild-type *covS* gene into MGAS315 Δ*covS* using the suicide plasmid p740-*covS* ^wt^, which was previously constructed to contain the *covS* gene and its flanking fragments from MGAS2221 [22]. The successful replacement of the *covS* ^1370T^ gene in MGAS315 with the wild-type *covS* gene in a *covS* ^wt^-knock-in strain (MGAS315 *covS* ^wt^) was confirmed by DNA sequencing. An MGAS315 *covS* ^wt^::p740-Δ*covS* strain from a single crossover between the *covRS* locus in MGAS315 *covS* ^wt^ and p740-Δ*covS* was selected by chloramphenicol, and the insertion of p740-Δ*covS* at the *covS* locus in the strain was confirmed by PCR.

### *In trans* complementation of MGAS315, MGAS6180, and MGAS2221 Δ*covS with covS* ^wt^, *covS* ^1370T^ and *ropB* ^wt^

The plasmids, pDCBB-*covS* ^wt^ and pDCBB-*covS* ^1370T^ containing the wild-type *covS* gene from MGAS2221 and *covS* ^1370T^ from MGAS315 for *in trans* expression, respectively, were constructed as follows. The *covS* ^wt^ and *covS* ^G1370T^ genes were amplified from MGAS2221 and MGAS315, respectively, using Phusion DNA Polymerase (New England Biolabs, Ipswich, MA) and primers 5’-GGGGACAAGTTTGTACAAAAAAGCAGGCTTCGTGAGAAATAAGTCATATGGA-3’ and 5’-GGGGACCACTTTGTACAAGAAAGCTGGGTCAATGCCTTAAGCTACTCTAA-3’. The underlined sequences were *attB* sequences for the BP clonase reaction. The PCR products were cloned into the donor vector pDONR221 using the BP clonase, yielding pDONR221-*covS* ^wt^ and pDONR221-*covS* ^1370T^. The *covS* ^wt^ and *covS* ^G1370T^ genes in these plasmids were transferred into pDCBB [16] by LR clonase-catalyzed reactions of these plasmids with pDCBB-RFA [42], yielding pDCBB-*covS* ^wt^ and pDCBB-*covS* ^1370T^. pDCBB-*ropB* was similarly constructed using the *ropB* gene amplified from MGAS2221 with primers 5’-GGGGACAAGTTTGTACAAAAAAGCAGGCTTCAACTAGGAAGGCTTGACAT-3’ and 5’-GGGGACCACTTTGTACAAGAAAGCTGGGTGCGTTAGCTTTTTAAAGAGCC-3’. The underlined sequences in these primers were the *attB* sequences. pDCBB-*covS* ^1370T^, pDCBB-*covS* ^wt^, pDCBB-*ropB*, or pDCBB was introduced into MGAS2221 Δ*covS*, MGAS315, and/or MGAS6180 by electroporation, and transformants were selected by chloramphenicol and are listed in Table 1.

### Quantitative RT-PCR analyses

Each GAS strain was grown at 37°C (5% CO_2_) in THY to OD_600_ of 0.2. Total RNA was isolated from GAS, as described previously [43]. RNA was converted into complementary DNA using the All-in-One First Strand cDNA Synthesis Kit from GeneCopoeia (Rockville, MD). Quantitative real-time PCR assays were performed using the All-in-One SYBR qPCR mix from GeneCopoeia. Primers used are: *hasA*, 5’-GGAGTTCAAACACAGATGCAATAC-3’ and 5’-CAAGGGAACGGTGAACGATAA-3’; *sse*, 5’-TGCAGCTAGTTCTCGTTCTTG-3’ and 5’-GGAAGCTTGGGTCATCTTGT-3’; *spyCEP*, 5’-CCGCTTGAACAGTCCTTGTA-3’ and 5’-CCTTCGATACGGTAGCCTTTAG-3’; *gyrA*, 5’-GCCGTTGGGATGGCAACTAACATT-3’ and 5’-TAACAAGGGCACCAGTCGGAAAGT-3’; *emm1*, 5’-GATCTTGCAGCAAACAATCCC-3’ and 5’-ATCTCTTCCTGCAACTTCCATT-3’; *emm3*, 5’-GCACCACAAGCAGGTACAA-3’ and 5’-CCGCTGTGAAGAATGGGTTAG-3’; and *emm28*, 5’-GAACGTCAAAGTCAACGAGAAATAG-3’ and 5’-GGCTTAAGCTCTTACGACTAGC-3’. All RNA samples were assayed in triplicates, and mRNA levels of each target gene were normalized first to the mRNA levels of the *gyrA* gene and then to those in MGAS315.

### Mouse infections

Five weeks-old, female CD-1 mice from Charles River Laboratories were used to compare various strains in virulence, neutrophil recruitment, skin invasion, and systemic GAS dissemination. Groups of 15 mice were subcutaneously inoculated with 0.2 ml of GAS in DPBS at OD_600_ of 1.0, which contained about 10^8^ colony-forming units (cfu). Five mice of each group were euthanized to collect skin samples for measurement of lesion size and neutrophil recruitment as described below, and the liver and spleen were also harvested to measure numbers of viable GAS. Other 10 mice of each group were monitored four times a day at 8:00 am, 12:00 am, 4:30 pm and 10:00 pm in the first 5 days after subcutaneous inoculation and twice a day (8:00 am and 4:30 pm) after day 5 for 14 days to determine survival rates. Humane endpoints described below were used in this survival study. All mice with subcutaneous GAS infection lose about 0.5 to 1 g on first 4 days (about 2% to 4% of total weight), have obvious ruffled fur on day 1, and have increased breathing rates. These symptoms were obvious even when mice were infected with MGAS6180 or with non-lethal doses of MGAS315. Thus, including these symptoms in a numerical scale for assessment of mortality in mouse as a humane endpoint would make it harder to accurately compare virulence of GAS strains. We found that the mobility of mice is a consistent indicator for mortality. The humane endpoints we used were either of the following two situations: 1) mice do not move when the lip of cage is moved or they move slowly in response to prodding and 2) mice have hind-limb paralysis. About two-thirds of non-surviving mice in this study were euthanized after they reached the humane endpoints. The euthanasia of mice was done with a gradual fill method at a displacement rate of 30% CO_2_ of the chamber volume per minute, as recommended in The 2013 American Veterinary Medical Association Guidelines. All the procedures except the euthanasia were done with mice under anesthesia through inhalation of 4% isoflurane. No analgesics were used during the course of infection. GAS loads in organs were determined by plating.

### Cytospin analysis

MGAS315 or MGAS6180 (0.1 ml of bacterial suspension in DPBS with an OD_600_ of 1.0) was injected with 0.9 ml air subcutaneously into female CD-1 mice. The mice were euthanized at different time after inoculation, and the air sac in each mouse was lavaged with 1ml of cold DPBS. An aliquot of the recovered lavage fluid was used to determine the total number of viable cells by trypan blue exclusion counts. A second aliquot of the recovered lavage fluid at appropriate dilution was used to prepare cytospin slides using a Shandon Cytospin Cytocentrifuge. The slides were stained using the Diff-Quik stain kit from Fisher Scientific. Neutrophils among 150 host cells on each cytospin slide were counted to determine the percentage of neutrophils, and the total numbers of neutrophils were calculated from the percentage data and the total counts of viable cells in the lavage samples.

### Cytokine measurements

Groups of 10 6-week old female CD-1 mice were inoculated with 0.2 ml of MGAS315 and MGAS6180 at OD_600_ of 1.0. Ten mice were euthanized with CO_2_ for each time point and each strain at different time points, and skin infection sites were collected and weighed. The skin samples were grinded in 2 ml DPBS in 15-ml conical tissue grinder (VWR catalog No. 47732–446) and centrifuged at 3500 rpm for 10 min. The supernatants were filtered through 0.2 μm filters and frozen until they were analyzed. Fifty μl of each sample was used to measure TNF-α, INF-γ, IL-6, and IL-1β using BD Cytometric Bead Array Mouse Th1/Th2/Th17 and IL-1β Cytokine Kits by following the procedures in the manufacturer’s manual.

### Other measurements and analyses

The SpeB activity in the supernatant of overnight GAS cultures was detected by using the casein plate assay as described previously [44]. Skin lesions were recognized by the boundary of inflammation after the skin around infection site was peeled off, and lesion sizes were measured by analyzing the lesion pictures using the area measurement tool of the Adobe Acrobat 9 software program (Adobe Systems Incorporated). Numbers of recruited neutrophils at infections sites in the skin were determined by the myeloperoxidase assay, as described previously [45]. Statistical analyses were performed using the Prism software program (Graph-Pad Software, Inc.) as follows: Survival data were analyzed using the Log-rank (Mantel-Cox) test, and multiple data sets were analyzed using One-way ANOVA Newman-Keuls Multiple Comparison Test. Other data of lesion sizes, neutrophil levels, cytokines, and GAS loads were analyzed using the two-tailed Mann-Whitney *t* test.

## Results

### RopB and CovS polymorphisms and diminished SpeB production in MGAS315 and MGAS6180

**Fig 1:**
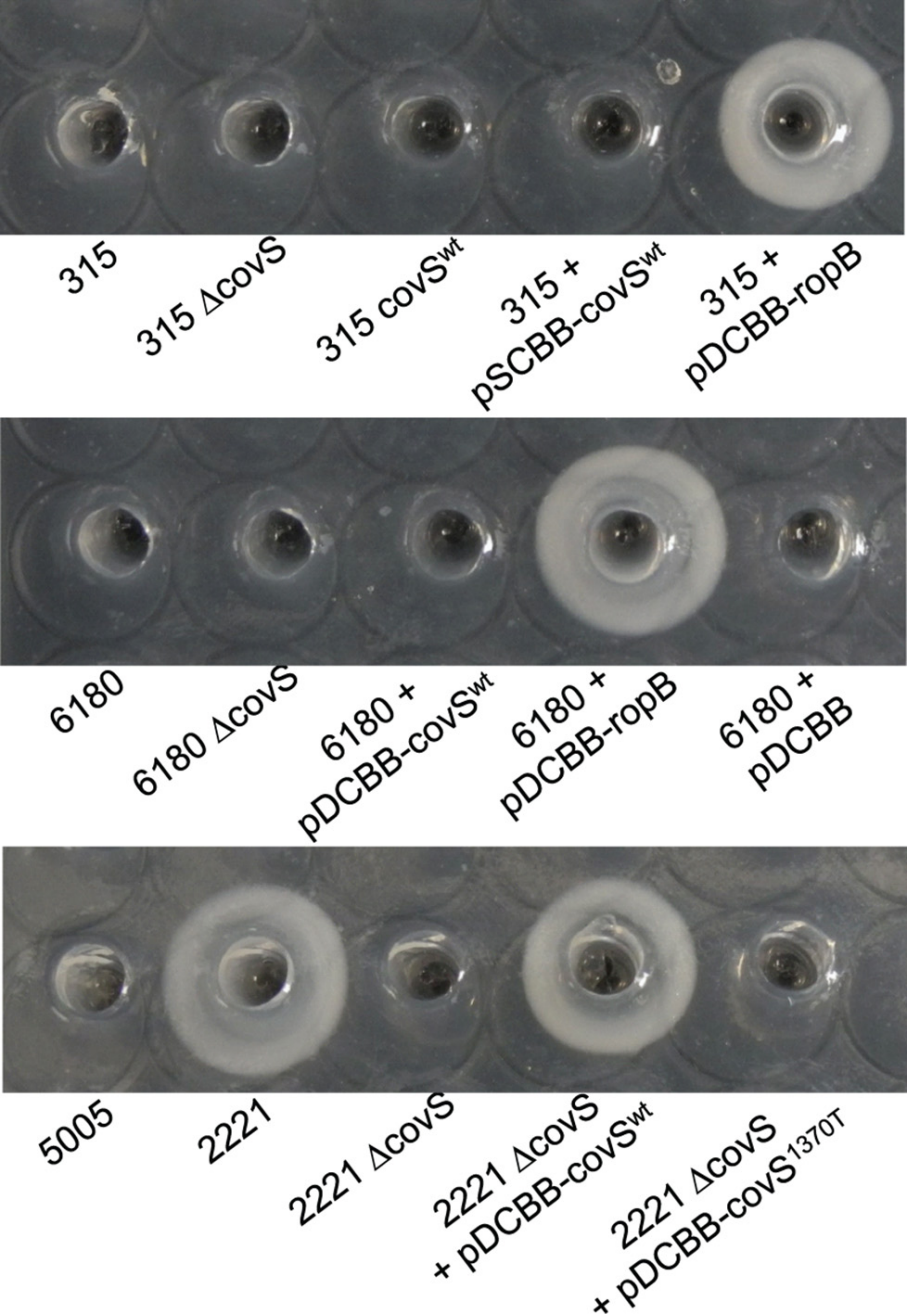
SpeB activity in the culture supernatant of GAS strains. MGAS315, MGAS2221, and MGAS6180 and their derivative strains were cultured overnight in THY, and SpeB activity in the supernatant of the cultures were detected by the casein hydrolysis plate assay.

GAS serotypes M3 and M28 are frequently associated with necrotizing fasciitis and puerperal sepsis, respectively [7,41]. According to the GAS genome databases [10,41,46], the CovS protein has G457V and E226G replacements in MGAS315 and MGAS6180, respectively, and the RopB/Rgg protein in MGAS315 and MGAS6180 has S103P and R11K mutations, respectively, compared with the CovS and RopB proteins of the M1 GAS strain SF370 [46]. Both MGAS315 and MGAS6180 had no detectable SpeB activity in the supernatants of their overnight culture, and *in trans* complementation with the MGAS2221 *ropB*/*rgg* gene, but not the MGAS2221 *covS* gene, restored SpeB production in both MGAS315 and MGAS6180 (Fig 1). These data indicate that the RopB mutations, but not the CovS mutations, were the basis for the SpeB activity-negative phenotype (SpeB^A-^) of MGAS315 and MGAS6180. The data for MGAS315 are consistent with the finding of Kappeler *et al*. on the critical role of the RopB/Rgg S103P mutation in causing the SpeB^A-^ phenotype in MGAS315 [47].

The CovS G457V or *covS* G1370T mutation did not cause the SpeB^A-^ phenotype in MGAS315 whereas complementation with the MGAS2221 *covS* ^wt^ gene, but not the MGAS315 *covS* ^G1370T^ gene, restored SpeB production by MGAS2221 Δ*covS* (Fig 1). These results suggest that the *covS* G1370T mutation present in MGAS315 would cause a SpeB^A-^ phenotype in MGAS2221 but not in MGAS315, implying that the regulation of SpeB expression in M1 GAS is different from that in M3 GAS.

### Comparison of MGAS315 and MGAS6180 in virulence, skin invasion, and systemic dissemination in subcutaneous infection of mice

**Fig 2:**
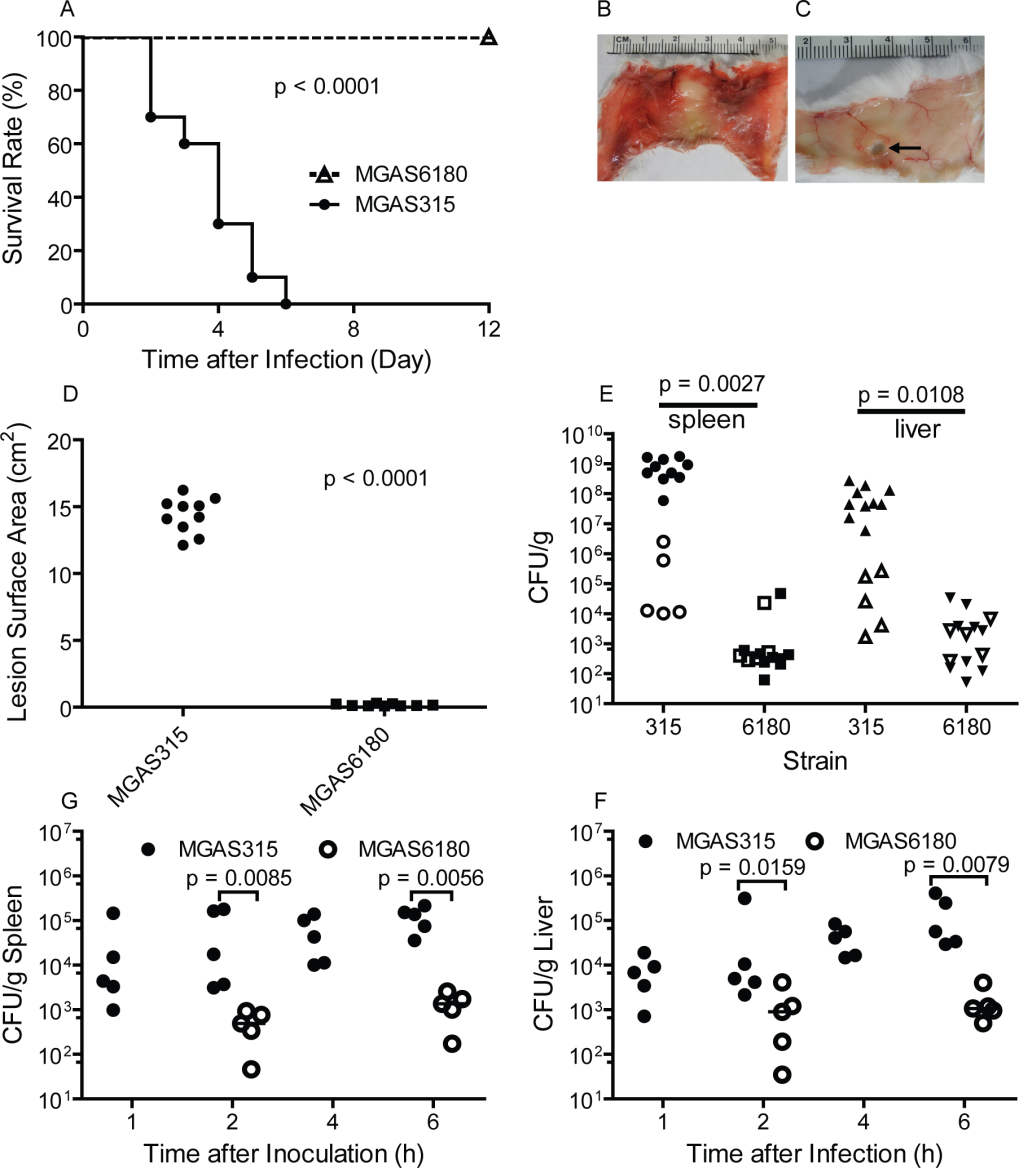
Phenotypic comparison of Serotype M3 strain MGAS315 and serotype M28 strain MGAS6180 in subcutaneous infection of mice. (A to E) Data in subcutaneous infection of mice with 0.2 ml MGAS315 (1.7 x 10^8^ cfu) and MGAS6180 (1.1 x 10^8^ cfu) suspensions at OD_600_ of 1.0: Survival curves of 10 mice per group (A); Representative inside-out images of skin infection sites for MGAS315 at the endpoint when death was eminent (B) and for MGAS6180 at the endpoint, day 12 after inoculation (C) in which the arrow indicates the hole left by infection-caused loss of the skin; Areas of skin lesions and holes at the endpoints for MGAS315 and MGAS6180 infections (D); Loads of MGAS315 and MGAS6180 in the spleen and liver at the endpoints (solid symbols) (E). Panel E also includes GAS loads at day 1 after inoculation of 8.0 x 10^7^ cfu MGAS315 and 1.1 x 10^8^ cfu MGAS6180 in a separate infection experiment (open symbols). (G and F) MGAS315 and MGAS6180 loads in the spleen (G) and liver (F) within hours after inoculation of 1.9 x 10^8^ cfu MGAS315 and 3.5 x 10^8^ cfu MGAS6180.

To determine whether subcutaneous infection of mice, a mouse model of necrotizing fasciitis, can distinguish the potential of invasive M3 and M28 GAS to cause soft tissue infections, subcutaneous infections of mice with MGAS315 and MGAS6180 were compared. All mice infected with MGAS315 did not survive whereas all mice inoculated with MGAS6180 did (p < 0.0001) (Fig 2A), demonstrating that the virulence of MGAS315 is much greater than MGAS6180 in the subcutaneous infection model. Inside-out pictures of skin infection sites show that MGAS315 spread almost to the whole skin at the end point when mice were dead or their death was imminent (Fig 2B) whereas MGAS6180 was restricted at inoculation sites where the fur had already fallen off by the end point, resulting in a whole in the skin that was peeled off at day 12 after inoculation (Fig 2C). MGAS315 caused an average skin infection area ± SD of 14.37 ± 0.42 cm^2^ at the end point, which was 85-fold greater than the average area of the holes in the skin caused by MGAS6180 (0.17 ± 0.02 cm^2^) (p < 0.0001) (Fig 2D). These results show that MGAS315 can severely invade the skin tissue whereas MGAS6180 cannot. Non-surviving mice infected with MGAS315 at the end point had (8.1 ± 1.8) x 10^8^ cfu/g tissue and (8.9 ± 2.7) x 10^7^ cfu/g tissue of GAS in the spleen and liver, respectively (Fig 2E, closed symbols). Mice infected with MGAS6180 were euthanized at day 12 after inoculation and had 5,495 ± 5,163 cfu/g and 7,180 ± 3,955 cfu/g of GAS in the spleen and liver, respectively (Fig 2E, closed symbols). Thus, mice can have MGAS6180 infection under control but cannot completely clear MGAS6180 bacteria, and, on the other hand, mice cannot restrain MGAS315 infections.

At day 1 after inoculation, mice infected with MGAS315 had (6.2 ± 4.8) x 10^5^ cfu/g and (9.4 ± 5.3) x 10^4^ cfu/g of GAS in the spleen and liver, respectively, which were 120- and 37-fold higher than the GAS load in the spleen (4864 ± 4484 cfu GAS/g) and liver (2484 ± 1182 cfu GAS/g) of MGAS6180-infected mice, respectively (Fig 2E, open symbols), suggesting that the two strains may have differential capability to disseminate. To further compare MGAS315 and MGAS6180 in systemic dissemination, GAS loads in the liver and spleen were measured within hours after GAS inoculation. MGAS315 was detected at 1 h after inoculation, and average GAS loads were changed from 3.4 x 10^4^ cfu/g at 1 hr to 1.2 x 10^5^ cfu/g at 6 h in the lung and from 7.8 x 10^3^ cfu/g at 1 h to 1.5 x 10^5^ cfu/g at 6 h in the liver (Fig 2E and 2F). MGAS315 loads were about 100-fold higher than MGAS6180 loads. Thus, MGAS315 indeed has a higher capacity than MGAS6180 to disseminate. Therefore, MGAS315 has a high capacity to invade skin tissue, to disseminate, and to cause lethal systemic infection whereas MGAS6180 causes a persistent infection with low bacterial loads and virulence.

### More severe evasion of neutrophil responses by MGAS315 than MGAS6180

**Fig 3:**
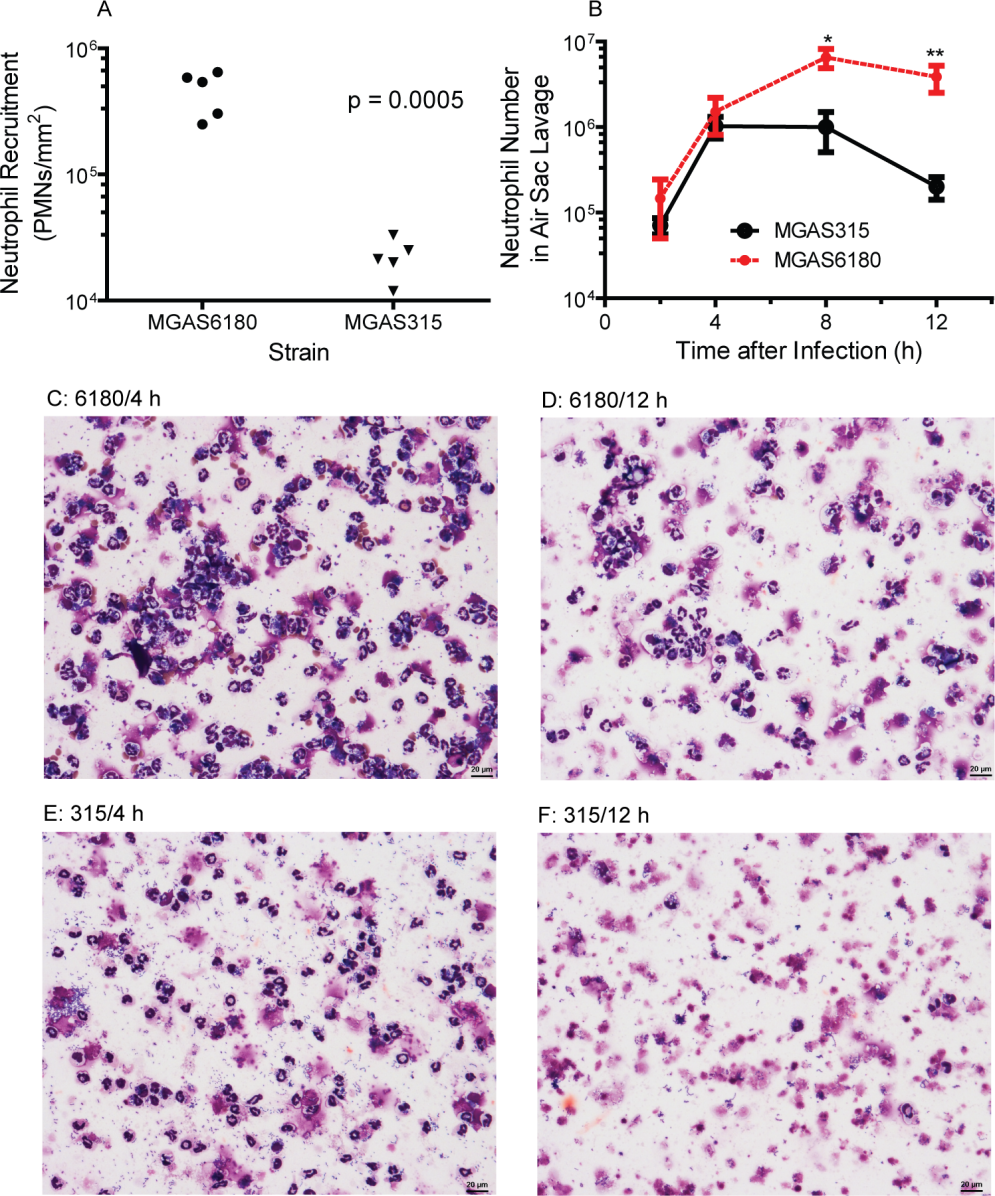
Comparison between MGAS315 and MGAS6180 in innate immune evasion. (A) Levels of neutrophils at skin infection sites in subcutaneous infections with 1.1 x 10^8^ cfu MGAS315 and 1.4 x 10^8^ cfu MGAS6180. (B-F) Data in the cytospin analysis of neutrophils infiltration in air sac infections with 6.6 x 10^7^ MGAS315 and 1.1 x 10^8^ cfu MGAS6180: Mean numbers ± error of viable neutrophils in the air sac lavages at the indicated times after GAS inoculation (B) and representative pictures of cytospin slides (C, no dilution of the lavage sample for cytospin; D-F, 2x dilution of the lavage samples for cytospin) comparing relative amounts of intact neutrophils versus cell debris at 4 h and 12 h in MGAS315 and MGAS6180 infections. *, P < 0.05, and **, P < 0.01, for the difference in neutrophil numbers between MGAS315 and MGAS6180 infections at 8 and 12 h, respectively.

The differences between the MGAS315 and MGAS6180 in GAS loads in the lung and liver at day 1 were about 1,000 fold less than those at the end point of the survival study. These results may be attributed to more efficient innate immune evasion by MGAS315 than MGAS6180. To test this possibility, we first measured neutrophil levels at MGAS315 and MGAS6180 sites in subcutaneous infections at 24 h after inoculation using the MPO activity assay. MGAS6180 sites had (4.6 ± 1.7) x 10^5^ neutrophils/mm^2^, which was 20-fold higher than those at MGAS315 sites [(2.2 ± 0.8) x 10^4^ neutrophils/mm^2^] (Fig 3A). Next, we used the cytospin analysis to measure neutrophil numbers in lavages of air sacs with MGAS315 and MGAS6180 at different time points after inoculation. Numbers of viable neutrophils in air sac lavages in MGAS6180 infection increased with time from 2 h to 8 h and decreased by 50% at 12 h compared with 8 h, and, in MGAS315 infection, neutrophil numbers at 2 and 4 h were similar to those in the MGAS6180 infection but decreased progressively at 8 and 12 h, being 6.5-fold and 19-fold less than those in MGAS6180 infection at 8 h and 12 h, respectively (Fig 3B). Consistent with these data, slides of the air sac lavages from MGAS315 infection showed many intact neutrophils and few cell debris at 4 h but lots of cell debris and few intact neutrophils at 12 h whereas the relative amount of intact neutrophils over cell debris from MGAS6180 infection did not have this dramatic change from 4 h to 12 h (Fig 3C–3F). These measurements indicate that MGAS315 may not only induce less neutrophil recruitment but also kills neutrophils more efficiently than MGAS6180.

### Suppression of TNF-α production at skin infection sites by MGAS315 but not by MGAS6180

**Fig 4:**
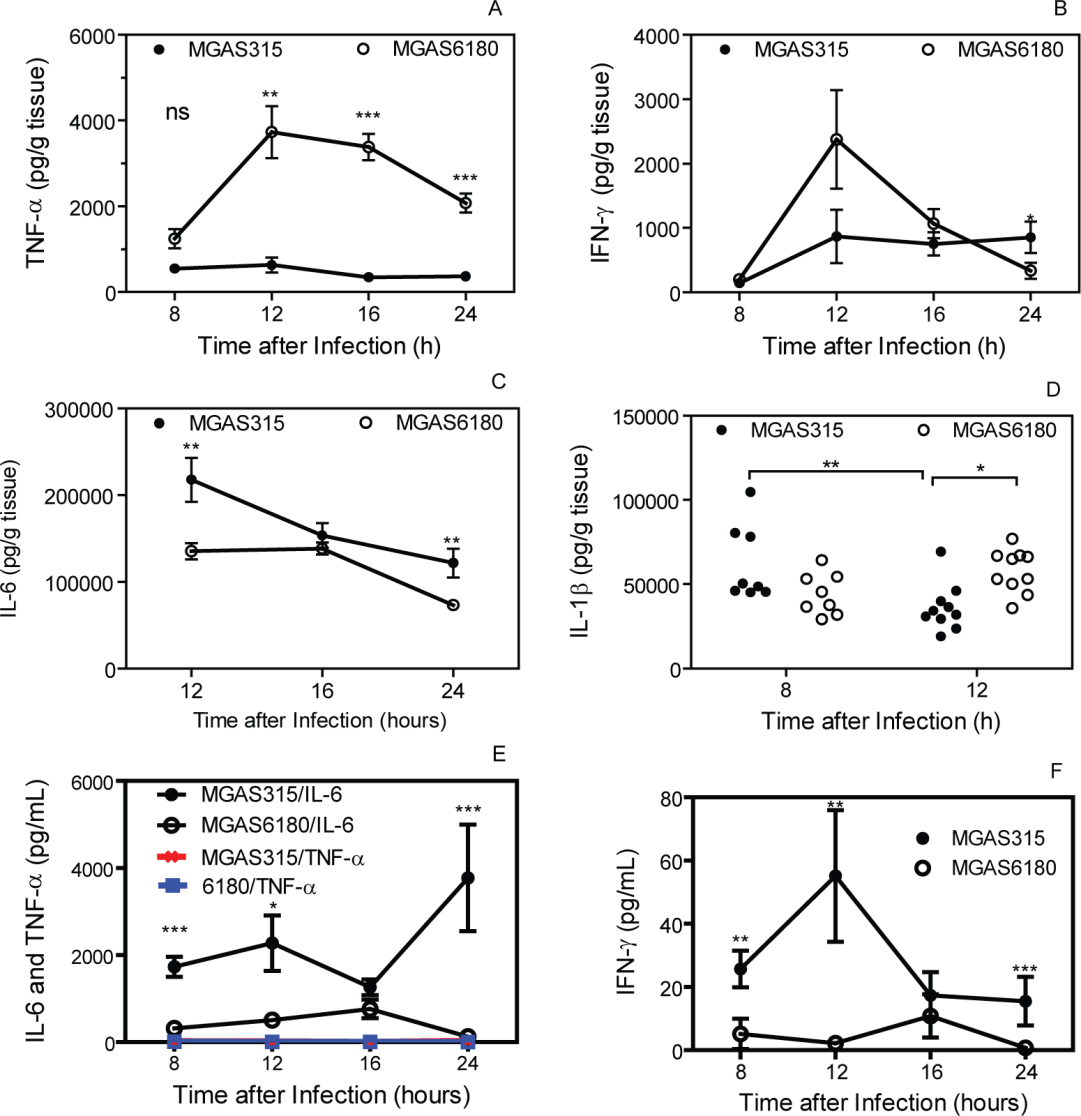
MGAS315 inhibits TNF-α production at skin infection sites. Presented are the levels of TNF-α (A), INF-γ (B), IL-6 (C), and IL-1β (D) at skin infection sites and levels of IL-6 and TNF-α (E) and INF-γ (F) in sera at the indicated time points after subcutaneous inoculation of 2.1 x 10^8^ cfu MGAS315 and 2.7 x 10^8^ cfu MGAS6180. Statistical analyses with the two-tailed Mann-Whitney *t* test for the cytokine levels between MGAS315 and MGAS6180 infections: ns, not significant; ***, P < 0.001; **, P < 0.01; and *, P < 0.05.

To search for additional innate immune evasion by MGAS315 in skin infection, cytokines TNF-α, INF-γ, IL-6, and IL-1β at skin infection sites of MGAS315 and MGAS6180 were measured. Levels of TNF-α at MGAS6180 infection sites were 2.27-, 5.8-, 9.9-, and 5.3-fold higher than those at MGAS315 infection sites at 8, 12, 16, and 24 h after GAS inoculation, respectively (Fig 4A). MGAS315 infection sites had higher levels of IL-6 than MGAS6180 sites, suggesting that the hypervirulent phenotype of MGAS315 is not due to a suppressed IL-6 response. MGAS315 infection sites had higher IL-1β than MGAS6180 sites at 8 h after inoculation but decreased at 12 h whereas levels of IL-1β at MGAS6180 sites were higher at 12 h than at 8 h, suggesting that MGAS315 may have a mechanism to reduce IL-1β responses. It appears that MGAS315 can substantially evade TNF-α-dependent immune responses in skin infection sites.

Both subcutaneous MGAS315 and MGAS6180 infections induced very low levels of TNF-α but MGAS315 induced significant levels of IL-6 and low levels of IFN-γ in blood (Fig 4E and 4F). Notably, MGAS6180 did not induce significantly levels of IL-6 and IFN-γ in blood, especially at 24 h after subcutaneous inoculation (Fig 4E and 4F). These data suggest that MGAS6180 either lead to bacterial loads in the blood and organs that are too low to induce systemic immune responses or has a mechanism to suppress systemic immune responses.

### Correction of the *covS* G1370T mutation in MGAS315

**Fig 5:**
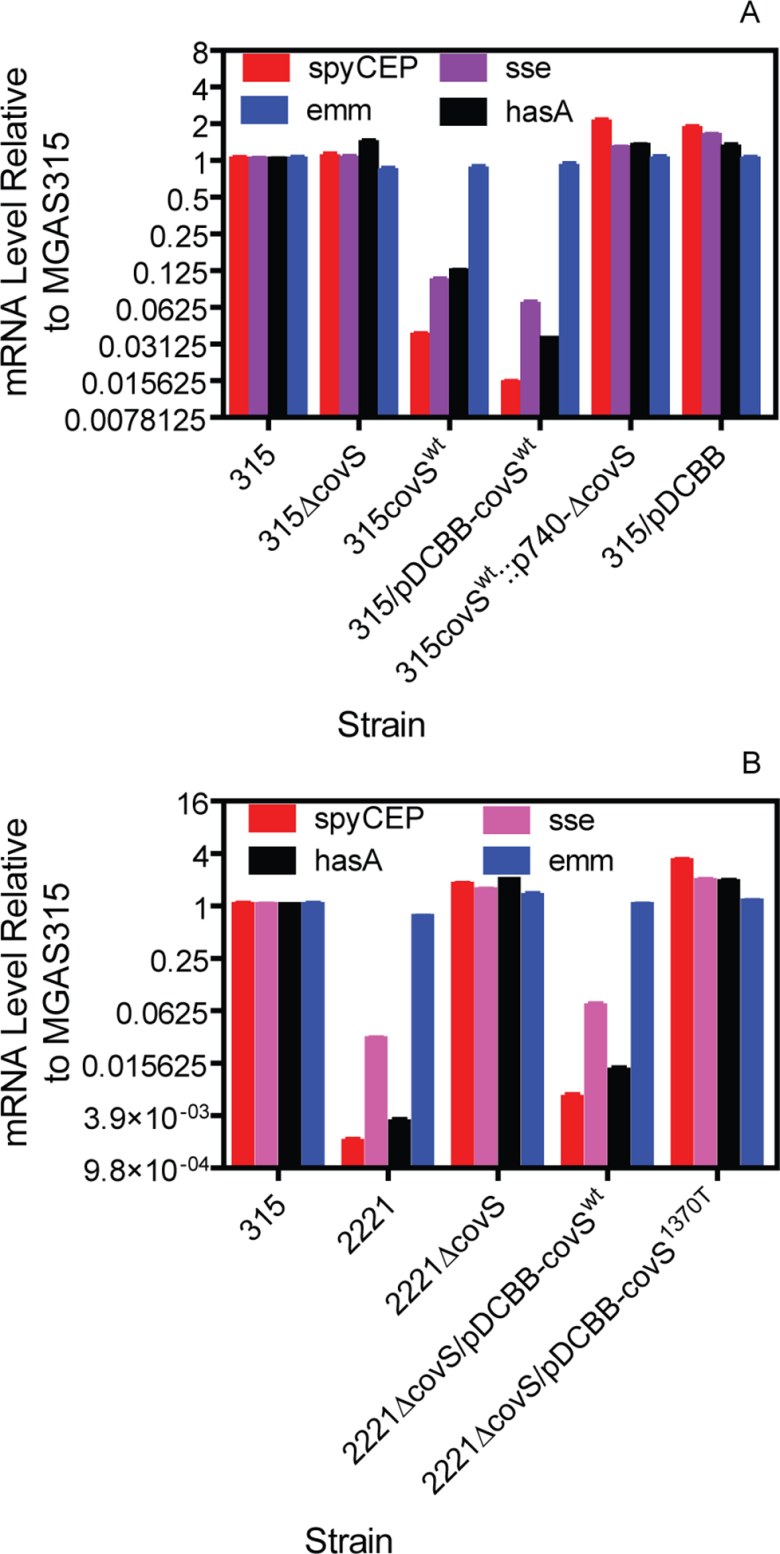
Effects of *covS* G1370T mutation on virulence gene expression. (A) Relative mRNA levels of *spyCEP*, *sse*, *hasA*, and *emm* in MGAS315, MGAS315 Δ*covS*, MGAS315 *covS* ^wt^, MGAS315/pDCBB-*covS* ^wt^, MGAS315::p740-Δ*covS*, and MGAS315/pDCBB. (B) Relative levels of *hasA*, *spyCEP*, *sse*, and *emm* transcripts in MGAS315, MGAS2221, MGAS2221 Δ*covS*, MGAS2221 Δ*covS*/pDCBB-*covS* ^wt^, and MGAS2221 Δ*covS*/pDCBB-covS^1370T^. All the mRNA levels were normalized first to that of *gyrA* in each strain and then to that of each gene transcript in MGAS315.

The phenotype of MGAS315 in subcutaneous mouse infection is similar to that of MGAS5005, which is caused by a natural null *covS* mutation [22]. It is possible that the *covS* G1370T mutation causes the hypervirulent phenotype of MGAS315. To test this possibility, we generated a derivative strain from MGAS315 in which the *covS* ^*1370T*^ gene was replaced by the *covS* gene from MGAS2221 (*covS* ^1370G^ referred as *covS* ^wt^). This replacement was achieved by first knocking out the mutated *covS* gene in MGAS315 to generate MGAS315 Δ*covS* and then by knocking in the MGAS2221 *covS* gene into MGAS315 Δ*covS* to generate MGAS315 *covS* ^wt^. These procedures did not introduce any foreign DNA sequences. In PCR analyses MGAS315 Δ*covS* mutants had the smaller *covS* PCR product than the parent strain MGAS315, and MGAS315 *covS* ^wt^ strains had a *covS* PCR product greater than its source strain, MGAS315 Δ*covS* (Data not shown). The *covS* ^1370T^-to-*covS* ^wt^ replacement in MGAS315 *covS* ^wt^ was confirmed by DNA sequencing. *In vitro* manipulation of GAS may lead to down-regulation of the *emm* gene that encodes the major virulence factor, the M protein, due to secondary mutations [48]. However, the MGAS315 Δ*covS* and MGAS315 *covS* ^wt^ strains had similar levels of the *emm*3 transcript with MGAS315 (Fig 5A), indicating that these strains had no alteration in the expression of the Mga regulon.

### Down-regulation of MGAS315 virulence genes by *covS* ^1370T^-to-*covS* ^wt^ replacement

To determine whether the *covS* G1370T mutation enhances the expression of CovRS-repressed virulence genes, levels of *hasA*, *spyCEP*, and *sse* transcripts in MGAS315, MGAS315 Δ*covS*, and MGAS315 *covS* ^wt^ at the mid-exponential growth phase were measured by real-time RT-PCR analysis. While the levels of these gene transcripts in MGAS315 were similar with those in MGAS315 Δ*covS*, MGAS315 *covS* ^wt^ had reductions in levels of *spyCEP*, *sse*, and *hasA* transcripts by 97%, 90%, and 88%, respectively, in comparison with MGAS315. MGAS315/pDCBB-*covS* ^wt^ in *in trans* complementation, but not MGAS315/pDCBB (vector control), had similar reduction in the transcription of these genes with MGAS315 *covS* ^wt^ (Fig 5A). To rule out the possibility that a secondary mutation during the construction of MGAS315 *covS* ^wt^ was responsible for the down-regulation of these virulence genes, p740-Δ*covS* was inserted in the *covS* locus in MGAS315 *covS* ^wt^ to yield a MGAS315 *covS* ^wt^::p740-Δ*covS* strain. This strain had increased *spyCEP*, *sse*, and *hasA* transcription to that in MGAS315. Thus, the *covS* G1370T mutation in MGAS315 enhances the expression of CovRS-controlled virulence genes. This conclusion is further supported by the complementation of MGAS2221 Δ*covS* with pDCBB-*covS* ^wt^ but not with pDCBB-*covS* ^1370T^. Levels of *hasA*, *sse*, *spyCEP* mRNA in MGAS2221 Δ*covS* were higher than those in MGAS2221 but similar to those in MGAS315 (Fig 5B). *In trans* complementation of MGAS2221 Δ*covS* with *covS* ^wt^, but not *covS* ^1370T^, reduced the levels of *hasA*, *sse*, and *spyCEP* mRNA to those in MGAS2221 while the levels of *emm* transcript were similar in all the strains as expected (Fig 5B).

### Attenuation of MGAS315 virulence by *covS* ^1370T^-to-*covS* ^wt^ replacement

**Fig 6:**
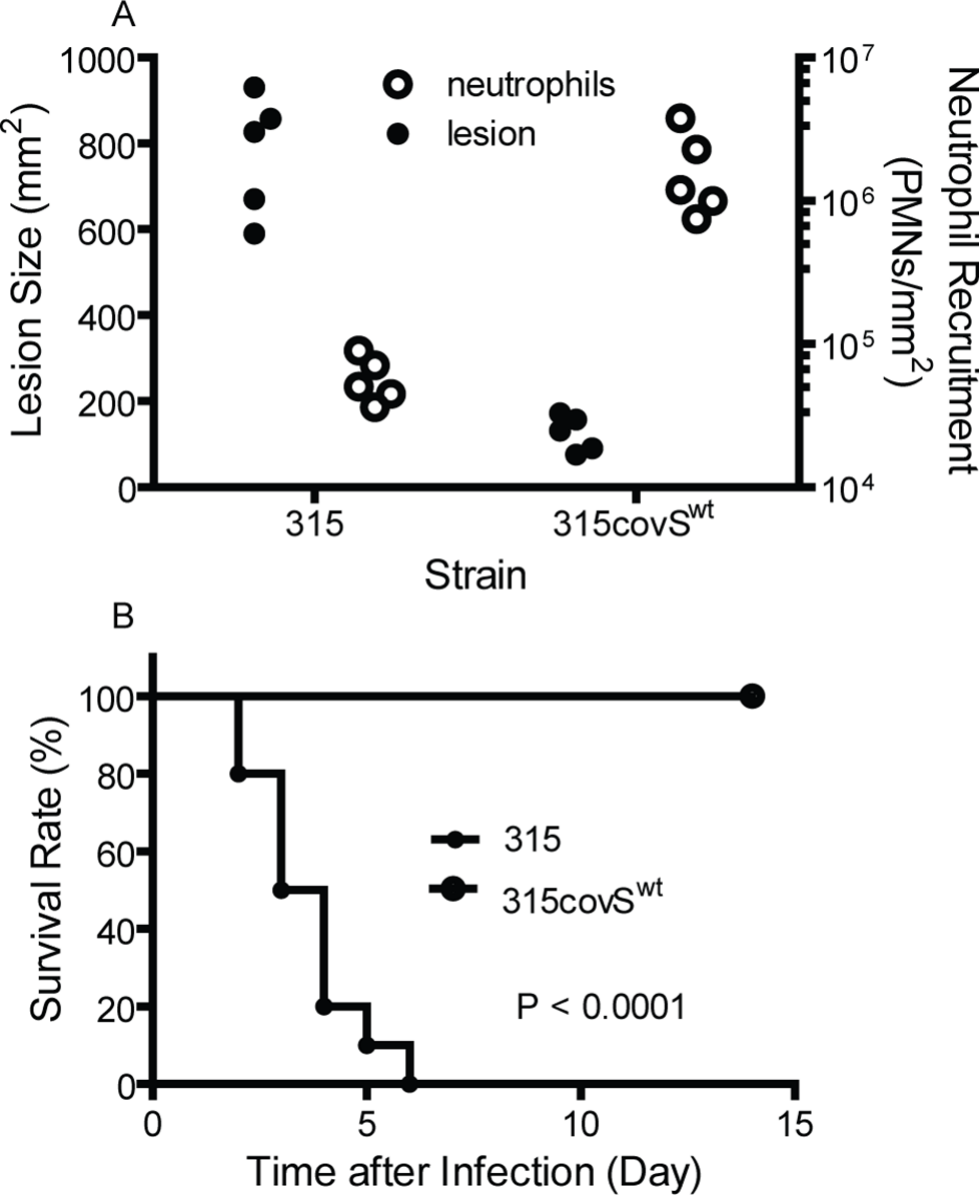
Replacement of *covS* ^1370T^ with *covS* ^wt^ in MGAS315 reduces skin invasion and virulence and enhances neutrophil recruitment in subcutaneous infection of mice. (A) Neutrophil levels and skin lesion sizes in MGAS315 and MGAS315*covS* ^wt^ infections. (B) Survival rates of mice infected with MGAS315 and MGAS315*covS* ^wt^. Inoculum: 1.3 x 10^8^ cfu MGAS315 and 1.4 x 10^8^ cfu MGAS315 *covS* ^wt^.

Since the replacement of *covS* ^1370T^ with *covS* ^wt^ reduced the expression of the multiple virulence genes of MGAS315, we hypothesized that the introduction of wild-type *covS* reduces the capacity of MGAS315 to invade soft tissue and evade innate immunity, resulting in attenuated virulence of MGAS315 *covS* ^wt^. Indeed, while MGAS315 caused an average lesion size of 775 ± 140 mm^2^ and induced (5.8 ± 2.2) x 10^4^ neutrophils/mm^2^ at 24 h after inoculation, MGAS315 *covS* ^wt^ caused small lesion size of 125 ± 42 mm^2^ and induced 30-fold more neutrophils [(1.8 ± 1.3) x 10^6^ neutrophils/mm^2^] (Fig 6A). All mice subcutaneously infected with MGAS315 *covS* ^wt^ survived but mice infected with MGAS315 did not (Fig 6B).

### MGAS315 Δ*covS* and MGAS315 *covS* ^*wt*^ have higher skin invasion capacity than MGAS6180 Δ*covS* and MGAS6180, respectively

**Fig 7:**
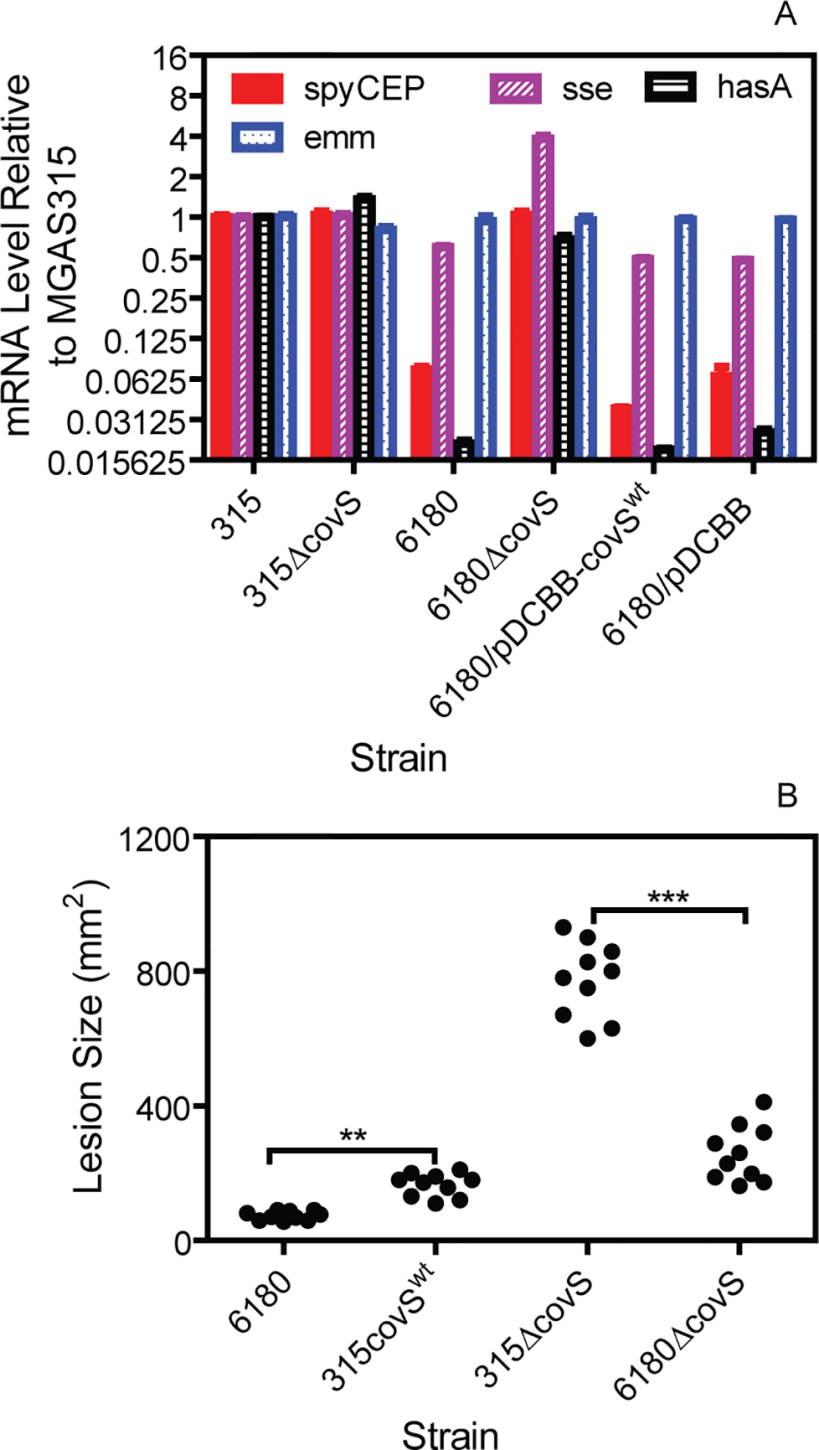
MGAS315 *covS* ^wt^ and MGAS315 Δ*covS* have higher skin invasion than MGAS6180 and MGAS6180 Δ*covS*, respectively. (A) Relative levels of *hasA*, *spyCEP*, *sse*, and *emm* transcripts in MGAS315, MGAS315 Δ*covS*, MGAS6180, and MGAS6180 Δ*covS*, MGAS6180/pDCBB-*covS* ^wt^, and MGAS6180/pDCBB. All the mRNA levels were normalized first to that of *gyrA* and then to that of each gene transcript in MGAS315. (B) Lesion sizes in subcutaneous infections of mice at 24 h after inoculation after inoculation with 1.3 x 10^8^ cfu MGAS315 *covS* ^wt^, 1.2 x 10^8^ cfu MGAS315 Δ*covS*, 1.4 x 10^8^ cfu MGAS6180, and 1.3 x 10^8^ cfu MGAS6180 Δ*covS*. Statistical analysis with the One-way ANOVA Newman-Keuls Multiple Comparison Test in panel B: **, P < 0.01 and ***, P < 0.001.

Since MGAS315 has a non-functional *covS* mutation, the distinct phenotype of MGAS315 and MGAS6180 in skin infection might be determined by the functionality of CovRS. To examine this possibility, we compared MGAS315 Δ*covS*, MGAS315 *covS* ^wt^, MGAS6180, and MGAS6168 Δ*covS* in the levels of *hasA*, *spyCEP*, *sse*, and *emm* transcripts and skin invasion. MGAS315 Δ*covS* and MGAS6180 Δ*covS* had similar levels of *hasA* and *spyCEP* transcripts with MGAS315 whereas MGAS6180 had much lower *hasA* and *spyCEP* transcription than MGAS315 (Fig 7A). *In trans* complementation of MGAS6180 with pDCBB-*covS* ^wt^ did not alter *hasA* and *spyCEP*, and *sse* transcription (Fig 7A). MGAS6180 had significant higher *sse* expression, and *covS* deletion increased *sse* expression by 5 fold (Fig 7A), confirming our previous data [42]. As expected, all the strains had similar *emm* expression. Thus, MGAS6180 and MGAS315 *covS* ^wt^ had similar low *hasA* and *spyCEP* expression whereas MGAS315 Δ*covS* and MGAS6180 Δ*covS* had similar high *hasA* and *spyCEP* expression. MGAS315 *covS* ^wt^ and MGAS315 Δ*covS* caused 2.2- and 3-fold greater skin lesion than MGAS6180 and MGAS6180 Δ*covS*, respectively (Fig 6B). Thus, MGAS315 derivative strains had greater capacity to invade soft tissue than MGAS6180 and MGAS6180 Δ*covS* in the presence and absence of functional CovRS, respectively.

## Discussion

The purpose of this study is to understand the basis for the frequent association of serotype M3 GAS with necrotizing fasciitis. We took a comparative approach to compare two sequenced GAS strains, M3 strain MGAS315, and M28 strain, MGAS6180, and their derivatives in subcutaneous infections. We present the following findings: I) In contrast with MGAS6180, MGAS315 has an extremely high capacity to invade soft tissue, evade neutrophil responses, disseminate and kill mice in subcutaneous infection; II) MGAS315 suppresses TNF-α production at skin infection sites; III) the *covS* G1370T mutation in MGAS315 critically contributes to its hypervirulent phenotype; and IV) M3 GAS has significantly higher soft tissue-invading capacity than M28 GAS with or without functional CovRS. These findings suggest that contemporary M3 GAS has a high capacity to evade neutrophil and TNF-α-dependent immune responses and to invade soft tissue, which is not possessed by GAS of many other serotypes, and that natural CovS polymorphisms maximize this soft tissue-invading capacity of M3 GAS to cause severe invasive infections.

Parallel comparison of invasive isolates of M3 and M28 serotypes in this study provides evidence for tissue tropisms of GAS of different serotypes. GAS isolates from necrotizing fasciitis are disproportionally those of serotype M3 [7] whereas M28 GAS is associated with puerperal fever [41]. Isogenic MGAS315 derivatives strains with and without functional CovRS display higher skin invasion than isogenic MGAS6180 strains without and with *covS* deletion, respectively. Thus, contemporary serotype M3 GAS appears to have an intrinsic capacity to invade soft tissues. Evasion of innate immune responses is one factor for this skin invasion capacity and thus a contributing factor for the association of M3 GAS with soft tissue infections. Additional virulence factors and genetic polymorphisms may also contribute to the invasiveness of M3 GAS. A phage containing the phospholipase A2 gene, *slaA*, is present in contemporary invasive M3 GAS strains but not in M3 isolates recovered before 1980s [10]. Deletion of *slaA* in MGAS315 reduces GAS colonization and persistence at the tonsils of monkeys and virulence in intraperitoneal infection of mice [49]. Whether SlaA critically contributes to skin invasion is not known.

MGAS315 *covS* ^wt^ still has the SpeB^A-^ phenotype, and *in trans* complementation of MGAS315 with wild-type *ropB* restores SpeB production, confirming the previous finding that the RopB S103P mutation in MGAS315 is responsible for the SpeB^A-^ phenotype [47]. MGAS6180 also has a SpeB^A—^causing RopB mutation. The attenuation of MGAS513 *covS* ^wt^ in virulence compared with MGAS315 indicates that RopB mutations alone do not critically contribute to the invasiveness of MGAS315. This conclusion is consistent with the previous finding that *ropB* depletion and polymorphisms that result in down-regulation of SpeB production actually reduce virulence [50]. However, SpeB^A—^causing RopB mutations may contribute to hypervirulence of invasive M3 GAS with certain *covRS* mutations. The combination of the CovS G457V and RopB S103P mutations in MGAS315 effectively causes the effects of a *covS* mutation in M1T1 GAS, the enhanced expression of multiple virulence genes and the suppression of SpeB production. The suppressed production of SpeB has been proposed to avoid the degradation of virulence factors [51,52]. Clinical M3 GAS isolates have frequent mutations of RopB/Rgg [50,53], and SpeB^A—^causing RopB mutations occur in both pharyngitis and invasive infection isolates [54]. It appears that RopB mutations in invasive M1 GAS are less frequent than those in invasive M3 GAS [53]. Interestingly, CovS^G457V^ could not restore SpeB production in MGAS2221 Δ*covS* whereas it had no detrimental effect on SpeB production in MGAS315/pDCBB-*ropB*. It is possible that the more frequent RopB mutations in clinical M3 GAS isolates might be due to the requirement of mutations in both CovS and RopB for better survival of M3 GAS against innate immune responses of hosts. We are testing this idea in a follow-up study.

One novel result in this study is that MGAS315 substantially inhibits the production of TNF-α compared with MGAS6180 at skin infection sites. TNF-α KO mice are more susceptible to GAS infection, and the enhanced susceptibility is independent of neutrophil recruitment but is believed to be due to the reduction in infiltration of inflammatory macrophages [55]. Evading the TNF-α response is likely a critical mechanism for the high capacity of MGAS315 to invade the skin tissue, which will be investigated in our follow-up study. The two strains also induced significant difference in IL-6 production at skin infection sites. However, MGAS315 induced higher levels of IL-6 than MGAS6180, indicating that MGAS315 does not target IL-6 response for achieving its hypervirulence. The two strains induced significant difference in IL-1β production at skin infection sites; however, the difference may not be a game changer for the following reasoning. First, both the strains induced high levels of IL-1β, and, second, the relative difference in IL-1β production changed with time during infection.

MGAS6180 is persistent at low levels in the liver and spleen. Interestingly, the presence of MGAS6180 in the organs during subcutaneous infection did not induce significant cytokine production in blood. This result is in sharp contrast with the potent local immune responses at skin infection sites of MGAS6180. It is not clear whether the lack of the systemic immune response is due to low bacterial loads or the presence of an active mechanism to suppress systemic immune responses. Persistence at low levels without induction of immune responses may be a trait of M28 GAS to colonize the vagina.

MGAS315 is a representative strain of the principal clone of M3 isolates that cause contemporary invasive infections in the U.S., Canada, Western Europe, and Japan [10], and its genome sequence has been used as a reference sequence for population-based genetic analyses [12,30,39]. This strain is highly virulent in intraperitoneal infection of mice [38]. We show in this study that MGAS315 is extremely invasive in skin infection of mice and that the CovS G457V mutation critically contributes to the high invasiveness of MGAS315. The critical contribution of the CovS G457V mutation to the high invasiveness is apparently the result of the G457V mutation-caused enhancement in the expression of multiple virulence genes. MGAS315 *covS* ^wt^ induced 30-fold more neutrophils than MGAS315. These results indicate that the CovS G457V mutation-mediated enhancement in the innate immune evasion plays a significant role in the high invasiveness. Miyoshi-Akiyam *et al*. found that a CovR missense mutation is responsible for the hypervirulence of an invasive M3 isolate [56]. These findings are consistent with those made by a number of groups for the M1T1 clone that is associated with the contemporary invasive GAS infections. Natural and mouse infection-derived *covS* missense and nonsense mutations and frame-shifting base(s) deletion and insertions of invasive M1T1 GAS up-regulate multiple virulence genes, inhibit SpeB production, and enhance virulence and skin invasion [19–21,31–37,45]. CovR mutations can also lead to the same hypervirulent M1T1 phenotype [36,37]. Neutrophils play a critical role in the selection of hypervirulent CovRS mutations of M1T1 GAS to enhance innate immune evasion and to survive better against innate immune responses [37].

The finding on the effect of the CovS G457V mutation on MGAS315 virulence is not merely a confirmation of the role of CovRS mutations in virulence, the knowledge that is primarily acquired from the studies on the M1T1 GAS subclone. Invasive M3 GAS isolates have significantly more CovS mutations than M3 pharyngitis isolates [12]. CovRS mutations are found in 49 of 95 invasive M3 isolates collected in Canada [39], and in half of 26 invasive M3 GAS strains isolated in Japan [53]. These frequencies are comparable to the frequencies of M1T1 GAS CovRS mutants among GAS isolates recovered from mice 3–4 days after inoculation of M1T1 GAS with wild-type CovRS [19,37]. Furthermore, whether invasive GAS isolates have CovRS mutations depend on where they are isolated [57]. These results suggest that patients with invasive M3 GAS infections have a mixture of GAS bacteria with and without CovRS mutations. In other words, patients with invasive M3 GAS infections from whom isolates with wild-type CovRS were obtained might also have CovRS mutants. These previous observations and our discussion are consistent with an idea that CovRS mutations arise during human infections with GAS carrying wild-type CovRS and are not transmissible [58]. Selection of CovRS mutations during infection and the experimental demonstration of the contribution of CovRS mutation to hypervirulence of M3 GAS indicate that, like emergence of invasive M1T1 GAS mentioned earlier, CovRS mutations are responsible for emergence of serotype M3 GAS with an invasive or hypervirulent phenotype during infections. Considering that clinical isolates of invasive M12 isolates usually lack CovRS mutations [53], experimental demonstration of the critical contribution of CovS mutations to hypervirulence of invasive M3 GAS is important to understand pathogenesis of invasive M3 GAS.

## Conclusions

In summary we have demonstrated that isogenic MGAS315 strains have higher capacity to invade skin tissue than isogenic MGAS6180 strains and that the CovS S457V mutation critically contributes to the hypervirulence and innate immune evasion of MGAS315. We conclude that contemporary invasive M3 GAS has a higher capacity to evade neutrophil and TNF-α responses at skin infection sites and to invade soft tissue than M28 MGAS and that the skin-invading capacity of M3 GAS is maximized by natural CovRS mutations.
